# The methodology for developing a prospective meta-analysis in the family planning community

**DOI:** 10.1186/1745-6215-12-104

**Published:** 2011-04-29

**Authors:** David K Turok, Eve Espey, Alison B Edelman, Pamela S Lotke, Eva H Lathrop, Stephanie B Teal, Janet C Jacobson, Sara E Simonsen, Kenneth F Schulz

**Affiliations:** 1 Department of Obstetrics and Gynecology, University of Utah School of Medicine, Salt Lake City, Utah, USA; 2 Department of Obstetrics and Gynecology, University of New Mexico, Albuquerque, New Mexico, USA; 3 Department of Obstetrics and Gynecology, Oregon Health & Science University, Portland, Oregon, USA; 4 Department of Obstetrics and Gynecology, University of Arizona, Tucson, Arizona, USA; 5 Department of Gynecology and Obstetrics, Emory University School of Medicine, Atlanta, Georgia, USA; 6 Department of Obstetrics and Gynecology, University of Colorado, Denver, Colorado, USA; 7 Department of Family and Preventive Medicine, Division of Public Health, University of Utah, Salt Lake City, Utah, USA; 8 Quantitative Sciences, Family Health International, Research Triangle Park, North Carolina, USA

## Abstract

**Background:**

Prospective meta-analysis (PMA) is a collaborative research design in which individual sites perform randomized controlled trials (RCTs) and pool the data for meta-analysis. Members of the PMA collaboration agree upon specific research interventions and outcome measures, ideally before initiation but at least prior to any individual trial publishing results. This allows for uniform reporting of primary and secondary outcomes. With this approach, heterogeneity among trials contributing data for the final meta-analysis is minimized while each site maintains the freedom to design a specific trial. This paper describes the process of creating a PMA collaboration to evaluate the impact of misoprostol on ease of intrauterine device (IUD) insertion in nulliparous women.

**Methods:**

After the principal investigator developed a preliminary PMA protocol, he identified potential collaborating investigators at other sites. One site already had a trial underway and another site was in the planning stages of a trial meeting PMA requirements. Investigators at six sites joined the PMA collaborative. Each site committed to enroll subjects to meet a pre-determined total sample size. A final common research plan and site responsibilities were developed and agreed upon through email and face-to-face meetings. Each site committed to contribute individual patient data to the PMA collaboration, and these data will be analyzed and prepared as a multi-site publication. Individual sites retain the ability to analyze and publish their site's independent findings.

**Results:**

All six sites have obtained Institutional Review Board approval and each has obtained individual funding to meet the needs of that site's study. Sites have shared resources including study protocols and consents to decrease costs and improve study flow. This PMA protocol is registered with the Cochrane Collaboration and data will be analyzed according to Cochrane standards for meta-analysis.

**Conclusions:**

PMA is a novel research method that improves meta-analysis by including several study sites, establishing uniform reporting of specific outcomes, and yet allowing some independence on the part of individual sites with respect to the conduct of research. The inclusion of several sites increases statistical power to address important clinical questions. Compared to multi-center trials, PMA methodology encourages collaboration, aids in the development of new investigators, decreases study costs, and decreases time to publication.

**Trial Registration:**

ClinicalTrials.gov: NCT00613366, NCT00886834, NCT01001897, NCT01147497 and NCT01307111

## Background

### Introduction

While well-conducted multi-center randomized controlled trials (RCTs) and meta-analyses are the gold standard for evidenced based medicine, they have weaknesses. Multi-center trials, managed by a single institution, require uniform IRB submission and study procedures from all participating institutions. Multi-center trials are expensive and give little flexibility to individual sites. Meta-analysis has become a common research tool to combine data from several trials and analyze them in the aggregate. However, traditional meta-analysis often suffers from the heterogeneity of included trials where each individual study may have tested a unique intervention and assessed a unique primary outcome. Other pitfalls associated with meta-analyses when trying to include all relevant data include selective reporting biases and unwillingness by researchers to share their data.

Prospective meta-analysis (PMA) is a collaborative research design in which individual sites perform randomized controlled trials (RCTs) and then contribute their data to the collaboration for the meta-analysis. The main advantage of PMA is the uniformity of the intervention (e.g. drug dosing) and data collection instruments for the primary and secondary outcomes. Additional advantages of the PMA methodology include the ability for individual sites to recruit only the required number of subjects for a site-specific primary outcome (in this case such as pain with IUD insertion or ease of IUD insertion) and to maintain autonomy over this smaller project. Combining results from each institution creates the power necessary to answer research questions requiring a larger number of subjects than many institutions could recruit individually. For example, only two sites are powered for the primary PMA outcome in our study.

PMAs allow flexibility at individual sites to maximize participant recruitment and the participation of sites with smaller research programs to contribute to a larger statistically powerful study. Additionally, PMA methodology promotes mentoring, and minimizes costs. Data are pooled with retention of stratification by study site and analyzed by the collaboration to determine the PMA primary outcome, while each site may analyze and publish its own data. Our project utilizes PMA methodology to assess whether misoprostol reduces the need for adjunctive measures to insert an intrauterine device (IUD) in nulliparous women. We aim to introduce this promising research methodology within the family planning research community.

### PMA as a Research Tool

Meta-analysis increases statistical power and provides more precise estimates of treatment effect by increasing sample size. While meta-analysis has become common in the medical literature, *prospective *meta-analysis is a relatively new methodology. In PMA, the specific interventions as well as the primary and secondary outcomes are determined before data from specific trials are published. In some PMAs, the research collaboration occurs before initiation of any study procedures. In this ideal model, co-investigators discuss study questions, study outcomes and study procedures prior to initiation of any of the site studies. In other PMAs, investigators have begun a study but determine they need more subjects than available at their site/s. In this case, prior to the completion of subject enrollment, they may create a PMA, inviting other investigators to collaborate with individual RCTs, but with some uniformity in study outcomes and procedures. In all cases, the PMA protocol must be finalized prior to the publication of results by any individual trial.

Once the research plan is developed and agreed upon by collaboration, each contributing site conducts its own RCT. The de-identified data, ideally including individual patient data, are pooled to perform the meta-analysis. PMA has been championed by experts in scientific evidence [1,2], and a study section of the Cochrane Collaboration is dedicated to this methodology [3]. PMA has been employed to address a variety of research questions, including reduction of cholesterol [4], treatment of hypertension [5], reduction of functional declines in elderly hospitalized patients [6,7], the role of genetics in disease outcomes [8,9], neonatal effects of epidural anesthesia [10], and childhood acute lymphoblastic leukemia [11]. The research effort described in this paper is the first to be registered with the Cochrane Collaboration [12].

### A PMA Example: Cholesterol Trials and Mortality

The PMA process is illustrated with the following example. Several large-scale trials were in progress to assess the impact of various statins (HMG-CoA reductase inhibitors) on a variety of cardiovascular outcomes. None of these trials was powered independently to assess cardiovascular and non-cardiovascular mortality [13]. The investigators of these studies agreed to contribute to a PMA addressing mortality. Included trials were required to utilize proper randomization procedures, to assign lipid modification as the main study variable, and to enroll at least 1000 patients for at least 2 years of follow-up. Data from 14 trials were combined resulting in 90,056 patients with a mean follow up of 5 years. The PMA demonstrated statistically powerful reductions in both all-cause and coronary mortality [9]. PMA answered an important clinical question that no individual center or trial could have resolved. This was achieved with greater statistical validity and ability to address secondary outcomes than a traditional meta-analysis for less cost and with greater investigator autonomy than a multi-center RCT. PMA may also be used for outcomes requiring smaller numbers of subjects with equal effectiveness [10].

### Advantages of PMA

Because all sites contributing to a PMA must agree on the intervention and the measurement of the primary and secondary outcomes, a primary flaw of conventional meta-analyses, the disparity across trials on interventions and measurement of outcomes, is avoided. Without the collaborative decisions of the PMA, independent sites could potentially generate incompatible data, making meta-analysis difficult and decreasing the statistical power of the primary outcome. In PMA, analysis of pooled results is more facile because of homogeneity of study outcome measures. A detailed description of this group's PMA protocol and data analysis plan is available in the Cochrane protocol document [14]. In addition, when individual patient data are available for all studies included in a meta-analysis, as occurs in a PMA, researchers have the ability to conduct stratified analyses and to control for potentially confounding variables. After agreeing upon critical aspects of protocols by those contributing to the collaboration, the PMA process benefits from its otherwise decentralized nature.

PMA, because of its decentralization, requires fewer resources than a multi-center RCT. When sites conduct their own trials, they may develop protocols that best fit their site and interests, including the availability of study participants, the development of unique secondary outcomes, the availability of institutional resources and the requirements of the site's institutional review board (IRB). Time to completion of a PMA site's study may be expedited by avoiding the individual site IRB difficulties that often occur in multi-center trials with centralized study protocols. Individual sites may begin recruiting participants and collecting study data once they have obtained IRB approval without waiting for approval from all sites. Data collection does not have to occur concurrently at all sites.

Decentralization encourages study population diversity improving the external validity of study results. Decentralization also lowers costs by avoiding centralized oversight. While economies of scale in a multi-center trial can be cost-effective in relation to placebo manufacture and printed materials, these savings may be quickly offset by the expense of site visits. Co-investigators can pursue smaller grants for individual sites rather than a pursuing a large, less readily obtainable grant for an unwieldy and costly multi-center trial. Internal validity is maintained since each site publishes the results of its own trial.

The PMA allows significant flexibility in trial design at individual sites. Other than the primary outcome, secondary outcomes and eligibility criteria agreed upon by the 6 sites, each site was free to design a trial that was different in some way. For example, although there is general agreement with the misoprostol dosing regimen of 400 mcg within the family planning community based on pharmacokinetic data [15,16], decisions about route of administration and timing of dosing were left to the individual sites. As a result of this design freedom, one site is testing at home vaginal or buccal administration of misoprostol 3 to 4 hours prior to insertion while a different site is using administration 90 minutes prior to insertion. The group of collaborators had two in-person meetings and many email communications before all members of the collaborative group agreed on the final PMA protocol.

PMA methodology also allows several investigators from each site to conduct the study and to qualify for authorship of individual site publications. In contrast, in multi-center RCTs, authorship is commonly limited to the principal investigators at each site. In PMA, individual publications focusing on unique secondary outcomes further knowledge in the field. Given publication requirements for promotion at academic institutions, PMA offers the advantage of expanding opportunities for research participation and authorship of peer-reviewed publications. The responsibility of authorship may optimize individual site study protocol execution (e.g. proper administration of medications, maximizing participant retention, collecting all data points) and motivate individual sites to conclude the project in a timely fashion.

PMAs may include both experienced researchers and new investigators with a shared research interest. Since individual PMA study sites may include a number of researchers, the spectrum of novice to experienced researcher may play a role and become study authors. Multi-center trials commonly utilize the most experienced research institutions with the greatest patient volumes, making it difficult for smaller sites with a less established research infrastructure to participate. By including both senior and new researchers, the PMA provides a vehicle for mentorship and enables multiple sites the opportunity to gain research experience and expertise. Participation in PMA provides smaller sites and less experienced researchers with well-developed protocols and the opportunity to recruit smaller numbers of participants. Broader use of PMA may encourage the family planning community to increase research capacity through collaboration.

### The Rationale for a PMA of misoprostol prior to IUD insertion in nulliparous women

IUDs are an ideal method of contraception for many women as they are effective, have few side effects or contraindications, require only a single act of motivation for many years of use and are highly rated among users [17,18]. While there is broad support for IUD use in nulliparous women [18-21], the actual insertion may be more difficult in nulliparous than in parous women. Several adjunct measures have been employed by healthcare providers to ease pain associated with IUD insertion [22]. Although misoprostol is commonly used to dilate the cervix for procedures similar to IUD insertion such as in first trimester abortion [23,24], hysteroscopy [25,26] and endometrial biopsy [27], there is a paucity of data regarding misoprostol use by providers to ease IUD insertion in nulliparous women [28,29].

The need for more definitive data to determine the efficacy of misoprostol use to ease IUD insertion in nulliparous women prompted the design of our PMA. Specifically, we wished to assess whether or not cervical preparation with misoprostol in nulliparous women affects the need for adjunctive measures for IUD insertion, such as use of ultrasound guidance, cervical dilation, anesthesia and/or analgesia. This research question is particularly amenable to PMA methodology because the outcome-difficulty with IUD insertion in nulliparous women-is relatively uncommon, requiring a large sample size.

## Methods

A preliminary PMA protocol was developed by an investigator and was submitted for review to the Prospective Meta-Analysis Methods Group of the Cochrane Collaboration. While the protocol was under review, potential collaborating sites were identified through a search of clinicaltrials.gov, by contacting known family planning researchers, and by presenting the PMA design and study plan at a national meeting of contraceptive researchers (Reproductive Health 2008, Minneapolis, MN). Only investigators able to enroll subjects to meet a pre-determined sample size joined the PMA group. The group of PMA co-investigators was formed to make collective decisions about a common protocol and to determine site responsibilities. Included in the group were a representative from each site, an epidemiologist (SS), and an expert in meta-analysis (KS). The project is coordinated through the University of Utah where the principal investigator conducts research. The contributors have an authorship agreement for final publication based on recruitment numbers.

Each site modified a template to create a unique protocol and independently coordinates its site's study, using its institutional IRB. De-identified data from each site will be included in the PMA database, assuming subjects are properly randomized, the same interventions are examined, and outcome data on primary and secondary endpoints are included. Each individual trial is registered on clinicaltrials.gov, follows CONSORT guidelines [30] and will be published independently. If other sites not currently participating in this collaboration meet the above criteria *and *have not published results, they will be invited to join the PMA. If possible, the PMA will review study protocols prior to implementation.

Prior to initiating the PMA, we searched computerized databases to assure that no published studies already met the requirements of this PMA. We searched for ongoing trials on the WHO International Clinical Trials Registry Platform (ICTRP) - http://apps.who.int/trialsearch/; National Institutes of Health (NIH) - randomized trial records (ClinicalTrials.gov); as well as the metaRegister of Controlled Trials - http://controlled-trials.com/mrct. With no date or language restrictions, we searched for published trials on PubMed, the Cochrane Central Register of Controlled Trials (CENTRAL), POPLINE, and EMBASE. We searched reference lists of retrieved titles for previously unidentified studies and attempted to identify unpublished trials by contacting experienced family planning researchers. In addition, we annually review the proceedings of conferences with a focus on contraception to become aware of any ongoing or future trials on the subject.

The primary outcomes of our PMA are: 1) To compare the ability to insert IUDs without the use of adjunctive methods (use of ultrasound guidance, cervical dilation, placement of a paracervical block and/or use of additional pain medication) in nulliparous women who were randomly assigned to misoprostol 400 mcg or placebo by any route of administration within 24 hours of IUD insertion, 2) to compare pain scores between the two groups using a 100-mm validated visual analogue scale, and 3) to compare providers' rating of ease of insertion using a 100-mm validated visual analogue scale. The sample size necessary to detect a difference between a 10% need for adjunctive measures for IUD insertion in the placebo group and 2% need in the misoprostol group is 208 patients in each group assuming a 1:1 ratio of treatment assignment, α = 0.05, β = 0.10 with 90% power. This would require 416 patients total.

Upon completion of the study at each site, individual patient data will contribute to the final meta-analysis using criteria outlined in the Cochrane Handbook for Systematic Reviews of Interventions [31]. Individual trial sites are encouraged to mention in publication of their individual results that their data are being contributed to this PMA. The coordinating site for the PMA final analysis is the University of Utah as agreed upon when establishing the collaboration and noted in the Cochrane Protocol document [14]. In addition, specific subgroup analyses are delineated in the Cochrane Protocol document and include timing of insertion relative to menses, type of IUD inserted, and variations in misoprostol timing and administration [14].

## Results

Six sites were identified and agreed to collaborate on the PMA. The study sites, their representative to the collaboration, target recruitment goals, and primary outcome to be assessed at that site are listed in Table 1. Three in-person meetings with attendees from all sites have been held over two years as well as a number of electronic communications. Through this process a PMA protocol and database were created and agreed upon by all 6 sites. Sites have benefitted from the use of shared resources: the study protocol, consent forms, data collection sheets, and study database have been shared between sites. Each site has modified the documents as needed to conduct its individual trial. Sharing of resources reduces the time commitment of co-investigators and ensures uniform data collection and entry for primary and secondary outcomes of the PMA. In addition, a central pharmacy is used by several sites to produce the randomized sequences of misoprostol and placebo pills, which are identical in appearance, taste, and odor. Especially important in a meta-analysis that relies on individual patient data, the use of a similar database for all sites will save considerable effort in determining the final results of the PMA.

**Table 1:** Collaborative Group with Site-specific Primary Outcome and Sample Size

Institution	Primary investigator	Route & timing* of misoprostol administration	Site-specific primary outcome	Target recruitment
Emory University School of Medicine	Eva Lathrop	buccal 2 hours prior	Ability to have the IUD inserted without the use of ancillary measures including mechanical dilation of the cervix, placement of paracervical nerve block, or using abdominal ultrasound for guidance.	75

Oregon Health & Science University	Alison Edelman	Buccal, 90 minutes	Patient perceived pain on 100-mm visual analogue scale (VAS;anchors:0 = none,100 mm = worst imaginable)	40

University of Arizona	Pamela Lotke	Vaginal or buccal 2 hours prior	Patient perceived pain on 100-mm visual analogue scale (VAS;anchors:0 = none,100 mm = worst imaginable)	60

University of Colorado	Stephanie Teal	Sublingual, 2 hours prior	Able to have the IUD inserted in a standard fashion without the ancillary measures of mechanical dilation of the cervix, placement of paracervical nerve block, or using abdominal ultrasound for guidance.	150

University of New Mexico	Eve Espey	Buccal, 2 hours prior	Patient perceived pain on 100-mm visual analogue scale (VAS;anchors:0 = none,100 mm = worst imaginable)	80

University of Utah	David Turok	Buccal or vaginal 2-4 hours prior	Operator perceived ease of insertion (based on a 100-mm visual analogue scale -anchors: 0 _ extremely easy, 100 mm_impossible).	100

*"timing" reflects number of minutes misoprostol will be administered prior to IUD insertion

All six sites have IRB approval for this project. As of April 22, 2011, three sites have completed enrollment and are at various stages of reporting results. The remaining three sites have begun enrollment and are proceeding with data collection. Individual sites have registered their trials at the National Institutes of Health Clinical Trials Registry (ClinicalTrials.gov identifiers# NCT00613366, NCT00886834, NCT01001897, NCT01147497 and NCT01307111). The final PMA will be published in the Cochrane Collaboration.

## Discussion

The adoption of PMA methodology by the family planning community may assist in answering research questions that require a larger sample size while avoiding some of the limitations of a traditional meta-analysis. PMA provides mentorship to new investigators and gives multiple sites, including those with less research infrastructure, the opportunity to participate in a trial. Broader use of PMA has the potential to promote more rapid and effective growth of the Family Planning research community, encouraging the testing of more hypotheses in well-designed clinical trials.

A primary aim of our PMA is to create a network that will foster novel research programs, particularly at sites with less research experience. While some sites are more likely to fulfill their enrollment projections, a site falling short of its goal will not derail the overall process and can still contribute the data that were collected to the PMA. The PMA can still reach the overall target number of subjects by enrolling additional participants at other sites. By identifying the outcomes of interest prior to individual study design, the PMA allows generation of the same specific measured endpoints from the study sites. Besides ease of analysis of these compatible data sets, the PMA methodology increases statistical power and allows smaller study sites to pool data. Flexibility is integral to the PMA process.

The current trials have been funded by small budgets supported by the hosting sites. A multi-center trial examining the same outcomes would have required much greater funding which typically requires considerable time to obtain. The small amount of funding for our PMA may ultimately yield a significant impact in a shorter time frame.

A benefit of registering a PMA with the Cochrane Collaboration is the review of the PMA protocol by some of the world's most experienced clinical trial researchers. For studies in the family planning field, this review is conducted by the Fertility Regulation Group of the Cochrane Collaboration http://www.fertility-regulation.org/. Continued use of the Cochrane Collaborative for this function will improve Family Planning research and place our research community in the vanguard of evidenced-based medicine.

## Conclusions

The six sites involved in our PMA collaboration have employed this research method to answer an important patient care question while improving research community cooperation and maintaining site autonomy. The process of developing this PMA has strengthened the collaborating research sites by fostering protocol development, reducing costs, aiding new investigators, and increasing statistical power to address the use of misoprostol prior to IUD insertion in nulliparous women. Answering a question of such broad importance to U.S. women will be the significant result of several sites' participation in the PMA. Future use of PMA methodology in the Family Planning research community could be helpful in addressing other important research questions such as the ideal technique for postpartum IUD insertion, cervical preparation for second trimester abortion, and approaches to improve the uptake of highly effective long acting methods of contraception. Having gained familiarity with PMA design, development and execution, this collaborative group is uniquely poised to expand the use of PMA to address these important research questions.

## List of Abbreviations

PMA: prospective meta-analysis; RCT: randomized controlled trials; IUD: intrauterine device; IRB: institutional review board; VAS: visual analogue scale; ICTRP: International Clinical Trials Registry Platform; NIH: National Institutes of Health.

## Competing interests

David Turok has received product research support from Duramed Pharmaceuticals, Inc. for research on the copper IUD for emergency contraception, has served on a Scientific Advisory Board for Bayer Women's HealthCare, and has served as a speaker and clinical trainer for the Implanon device for Schering-Plough.

Stephanie Teal has served on a Scientific Advisory Board for Bayer Healthcare, which manufactures an IUD, and as an Implanon clinical trainer for Schering-Plough.

Pamela Lotke has served as a speaker and clinical trainer for the Implanon device for Schering-Plough, and has served on an International Advisory Board for Bayer Healthcare.

The remaining author(s) declare that they have no competing interests: Eve Espey, Alison Edelman, Eva Lathrop, Janet Jacobson, Sara Simonsen, and Kenneth Schulz.

## Authors' contributions

DT is the central coordinator of the PMA. DT, AE, PL, EL, EE, ST are the principal investigators at their respective sites. KS conceived of the idea to apply PMA methodology to address this research question and provided technical assistance in the development of the PMA protocol. SS directed the development of the statistical analysis. All authors have been involved in the PMA collaboration, participated in the writing of this manuscript, and read and approved the final version.
